# Advancing the application of systems thinking in health: provider payment and service supply behaviour and incentives in the Ghana National Health Insurance Scheme – a systems approach

**DOI:** 10.1186/1478-4505-12-35

**Published:** 2014-08-05

**Authors:** Irene A Agyepong, Geneieve C Aryeetey, Justice Nonvignon, Francis Asenso-Boadi, Helen Dzikunu, Edward Antwi, Daniel Ankrah, Charles Adjei-Acquah, Reuben Esena, Moses Aikins, Daniel K Arhinful

**Affiliations:** 1 Department of Health Policy Planning and Management (HPPM), University of Ghana School of Public Health, P.O. Box LG 13, Legon, Accra, Ghana; 2 National Health Insurance Authority, No. 36, 6th Avenue, Opposite AU Suite, Ridge Residential Area, Accra. Private Mail Bag, Ministries, Accra, Ghana; 3 Private Consultant, P.O. Box DS 331, Dansoman Estates, Accra, Ghana; 4 Ghana Health Service Greater Accra Regional Health Directorate, P.O. Box 184, Accra, Ghana; 5 Korle-Bu Teaching Hospital, P.O. Box 77, Accra, Ghana; 6 Ghana Health Service, Policy, Planning, Monitoring and Evaluation Division (PPMED), Private Mail Bag, Ministries, Accra, Ghana; 7 Noguchi Memorial Institute for Medical Research, University of Ghana, P.O. LG 581, Legon, Accra, Ghana

**Keywords:** Complex adaptive systems, Ghana, Incentives, Provider payment, Supply, Universal health coverage

## Abstract

**Background:**

Assuring equitable universal access to essential health services without exposure to undue financial hardship requires adequate resource mobilization, efficient use of resources, and attention to quality and responsiveness of services. The way providers are paid is a critical part of this process because it can create incentives and patterns of behaviour related to supply. The objective of this work was to describe provider behaviour related to supply of health services to insured clients in Ghana and the influence of provider payment methods on incentives and behaviour.

**Methods:**

A mixed methods study involving grey and published literature reviews, as well as health management information system and primary data collection and analysis was used. Primary data collection involved in-depth interviews, observations of time spent obtaining service, prescription analysis, and exit interviews with clients. Qualitative data was analysed manually to draw out themes, commonalities, and contrasts. Quantitative data was analysed in Excel and Stata. Causal loop and cause tree diagrams were used to develop a qualitative explanatory model of provider supply incentives and behaviour related to payment method in context.

**Results:**

There are multiple provider payment methods in the Ghanaian health system. National Health Insurance provider payment methods are the most recent additions. At the time of the study, the methods used nationwide were the Ghana Diagnostic Related Groupings payment for services and an itemized and standardized fee schedule for medicines. The influence of provider payment method on supply behaviour was sometimes intuitive and sometimes counter intuitive. It appeared to be related to context and the interaction of the methods with context and each other rather than linearly to any given method.

**Conclusions:**

As countries work towards Universal Health Coverage, there is a need to holistically design, implement, and manage provider payment methods reforms from systems rather than linear perspectives, since the latter fail to recognize the effects of context and the between-methods and context interactions in producing net effects.

## Introduction

In 2005, the member states of the World Health Organization committed to Universal Health Coverage (UHC) [1]. Specifically, their commitment was to develop their health financing systems so that their citizens would have universal access to essential health services (defined in context) without having to suffer financial hardship in paying for them. Subsequently, in 2012, the United Nations General Assembly, in resolution A/67/L.36 of it is 67th session [2], called upon member states “*to value the contribution of Universal Health Coverage to achieving all inter related Millennium Development Goals with the ultimate outcome of health improvements…*”. For the purposes of this paper, we use UHC in our understanding of the essence of the World Health Organization definition of ensuring equitable universal access to a core package of essential health services without exposing people to undue financial hardship [3]. The details of this ideal will have to be defined in context; in all contexts it requires adequate resource mobilization as well as the equitable and efficient use of available resources. A critical part of this effort will be provider payment methods [4].

Provider payment methods refer to the mechanisms used to transfer funds from the purchaser to the providers of health services. These methods include line item and global budgets, salaries, capitation with or without fund holding for referral services, case-based payments, and fee for service with or without a fee schedule. The provider payment system, on the other hand, refers to the payment method combined with all supporting systems such as accountability mechanisms, management information systems, etc. Different provider payment methods create different provider behavioural incentives related to service supply since they have different effects on the relationship between provider income and costs for providing the service, as well as the relationship between activities and payment [4,5]. Apart from financial incentives, provider supply behaviour can be influenced by other factors, such as peer, professional, and client pressure, and factors internal to the provider such as value systems and ethics.

Also important to understanding incentives is that provider payment methods are introduced and implemented in health systems. Systems are made of separate but interdependent parts that interact with each other. Occurrences and outcomes within systems can only be fully understood by appreciating the relationship and interconnectedness between these parts [6-8]. Moreover, health systems are complex adaptive systems (CAS), constantly changing and governed by feedback. Intervening in one part of the system will almost always have ripple effects in other parts; they self-organize and adapt based on experience. To fully understand incentives in a CAS, it is important to apply a systems thinking perspective, studying the context in which the payment method has been introduced and the resulting interactions.

The current study therefore set out to explore, from a systems thinking perspective, the questions of: “What kinds of provider behaviour are occurring related to supply of health services to insured clients in Ghana?; What incentives might be driving the behaviour?; and What is the influence of provider payment methods on the incentives and the behaviour?” Our focus was on financial incentives for service supply behaviour related to the nationwide National Health Insurance (NHIS) provider payment methods of the Ghana Diagnostic Related Groupings (G-DRG) for services and itemized fees with a fee schedule for medicines. A per capita payment for primary care, which was an early pilot in one region at the time of the investigation, was not included in our research given the focus on nationwide payment methods. The focus on financial incentives was selected since behaviour motivated by financial incentives (real or perceived) was and remains a source of much debate, conflict, and concern within the Ghana NHIS and links closely with concerns about cost escalation and cost containment.

### Context: economic, socio-demographic, and health

After a long period of near stagnation, Ghana has seen rapid growth in its GNI from an estimated US$ 320 per capita in 2003, when the NHIS law was passed, to US$ 1,410 (Atlas method current US$) in 2011 [9]. It is traditionally an agricultural country with cocoa, timber, and gold as its main exports. Oil was discovered off-shore in 2006 and production in commercial quantities started in 2011. The amounts produced are still small, but the importance of oil to its economy is growing, and it has played some role in the evolution of its GNI per capita. The Consumer Price Index, which measures the percentage change over time in the general price level of goods and services in a country, has risen each year and has remained high over several decades. Annual averages since 2003, when the NHIS was established, have ranged between 10% to 27% [10,11] and the value of the cedi has declined against the dollar.

About half of Ghana’s estimated population of 26 million are below 15 years old. The majority of formal sector workers, with some exceptions, such as employees of some tertiary educational institutions, belong to the Social Security and National Insurance Trust (SSNIT) pension scheme. Based on the 2011 SSNIT annual report, 963,619 Ghanaians (about 4% of the total population) were active contributors [12]. Even if the figures are doubled to include formal sector workers who do not contribute to the SSNIT pension scheme, it would be reasonable to estimate that about 80% of Ghana’s adult working population is employed in the non-formal sector.

Mortality of children under 5 years has declined, albeit very slowly, from 155 per 1,000 live births in 1983 to 1987, to 80 per 1,000 live births in 2003 to 2008 [13]. Maternal mortality declined from 503/100,000 in 2005 to 451/100,000 in 2008 [14]. Shortages of skilled human resources have been and remain a problem. The World Health Report 2006 estimated that Ghana had 0.15 physicians and 0.92 nurses per 1,000 population. This compared with 2.14 and 9.95 in a high-income country like Canada and 0.77 and 4.08 in a sub-Saharan Africa middle-income economy like South Africa [15]. The country’s challenges, with inadequacies in infrastructure, equipment, tools, and supplies in the health sector, mirrors its human resource challenges. A little under 15% of the public sector budget is allocated to health and the per capita expenditure on health in 2013 was estimated at US$35 [16].

The Ghana Health Service, the service delivery agency of the Ministry of Health, employs most public sector providers. Others are employed by other public sector agencies with hospitals of their own, e.g., the Military, Police, and the Universities. Private service delivery is done by not-for-profit and self-financing (for-profit) providers. Mission clinics and hospitals under the umbrella of the Christian Health Association of Ghana (CHAG) form most private not-for-profit providers. The private self-financing sector is made of individual physician, dentist, and midwife practices, hospitals, laboratories, and pharmacies.

### The Ghana National Health Insurance Scheme (NHIS)

In September 2003, Ghana passed a national health insurance (NHI) law (Act 650) to replace public sector user fees introduced in the 1980s as part of structural adjustment programs. Though the term UHC was not used, the government’s stated policy objectives in setting up a NHI scheme show the principles of UHC. Both the original [17] and revised [18] NHI policy frameworks state: “*…the vision of government in instituting a health insurance scheme…. is to assure equitable and universal access for all residents of Ghana to an acceptable quality package of essential healthcare… every resident of Ghana shall belong to a health insurance scheme that adequately covers him or her against the need to pay out of pocket at the point of service use in order to obtain access…*”.

The Ghana NHIS is described in several publications [19-22]. The benefit package covers about 80% to 90% of the most common clinical conditions in Ghana. The NHI has a single payer arrangement through the NHI fund. The NHI fund is about 70% to 75% from a value added tax and 20% to 25% from formal sector SSNIT contributions, 2.5% of which are mandated to be transferred into the NHI fund monthly. A small amount of NHI financing comes from the annual premium, non-SSNIT contributors pay out of pocket and the registration fee paid by all subscribers.

### Nationwide NHIS provider payment methods

Ghana’s NHIS started implementation in 2004, with itemized billing with no standardized fee schedule for services and medicines as the provider payment method for public and private service providers. Each of the district schemes negotiated with their providers itemized fee rates for services, consumables, and medicines. In the face of growing concerns over inefficiencies such as random price variations for the same procedures and consumables, cumbersome billing and claim vetting procedures and cost escalation, the National Health Insurance Authority (NHIA) introduced, in 2008, a case based payment system known as the G-DRG for services and procedures, as well as standardized itemized fees for medicines based on a medicine list. Apart from a few modifications, this payment system has remained in use across Ghana in its original design since then.

Classically, the two core components of a DRG payment system are a patient classification system and a payment rate setting mechanism that takes into account the intensity of resources used to treat patients in a given DRG category to give cost weights or prices to the DRG [23]. The G-DRG is not a pure DRG system in that, although it has the patient classification system, it does not have cost weights and severity levels. It was designed, applied, and continues to be applied nationwide for all levels of care from the lowest (Community Health Planning and Services (CHPS) compounds) to the highest (teaching hospitals), to pay all accredited providers – public, quasi-government, and private – for inpatient and outpatient services. The tariffs reflect preceding charges rather than a precise or economic costing; capital and equipment costs are not included. The tariffs are classified into three broad groups of diagnoses, procedures/operations, and investigations. The calculated direct cost of the services for consumables and labour are uniform for related or similar diagnosis, procedures, and investigations irrespective of level of care.

Indirect or overhead costs comprising labour, vehicle maintenance and fuel, equipment and building maintenance, housekeeping, utilities, and general administrative and office expenses are calculated, increasing from the lower to the higher level of care. The rationale is that facilities at higher levels of care consume larger amounts of overhead inputs because of their size and higher fragmentation of services. The tariffs vary according to whether the facility is government, mission, or private to take into account the government subsidy, mainly for salaries but also some infrastructure, equipment, and overhead costs in the public and, to some extent, the private mission sector, as well as the zero subsidy in the private self-financing sector. The tariffs also vary by type of final service (inpatient or outpatient), type of intermediate service (laboratory investigations, imaging investigations, theatre, catering services), and specialty (obstetrics and gynaecology, medicine, surgery, child health, eye, ENT, and dental). Since some district hospitals have catering services and others do not, inpatient tariffs differ by district hospitals with catering services and those without [24].

The itemized fee schedule for medicines is based on a NHI medicines list (NHIML) that is periodically revised. Medicines can be dispensed by public and private provider facilities with an in house pharmacy/dispensary or by private community practice pharmacies accredited by the NHIA. Most community practice pharmacies, like other private self-financing services, are based in wealthier and peri-urban areas. Poorer rural communities rely on chemical sellers (lay people licenced by the Pharmacy Council to sell over the counter medicines). Some of these are also accredited by the NHIA. In theory, there is supposed to be a separation between prescribing and dispensing; in practice, it is not enforced.

Payment to providers for services and medicines was and remains retrospective. Providers file claims, which go through a vetting process in the NHIA district scheme offices or for the higher-level facilities such as teaching and regional hospitals in the computerized central claims processing office of the NHIA, before final payment. The claims processes of many provider and district scheme offices remain predominantly manual despite increasing computerization. There remain administrative capacity, human resource, technical, and other challenges that slow down the process and can reduce the final value of the reimbursements [25].

The first review of the G-DRG tariffs after introduction in 2008 occurred when the Minister for Health, in response to provider agitations, announced an interim upward adjustment of G-DRG tariffs effective 1 July 2011. The increments were calculated based on an analysis of the trends in medical inflation since 2008, when the G-DRG was first introduced. Inpatient tariffs were increased by 30%, primary outpatient care by 22%, diagnostic services by 22%, and secondary and tertiary outpatient care by 25%. In that same year (2011), the first formal review of the G-DRG was commissioned by the NHIA. Stallion & Milliman Consultants were engaged for this review whose objectives were to: “*simplify the fee system, increase transparency and ensure that the G-DRG developed were consistent with Ghana’s standards of treatment*” [26]. The review was completed in 2012 and resulted in a further upward adjustment in the rates for all the G-DRG and some changes in the G-DRG groupings with some removed or merged, or new ones developed. The overall average change in the G-DRG tariffs was about 26% above the rates that were set in July 2011. Implementation of the new tariffs started on 1st February 2013.

**Figure 1:**
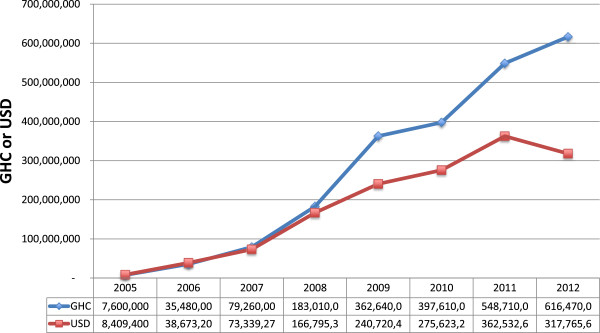
Total value of all NHIS claims (medicines, services, inpatient, and outpatient) reimbursed.

The first review of the NHIML and prices was in October 2009, the second in March 2011, and the third in July 2013. Data on the percentage increase in tariffs for the first and second review could not be found. However, for July 2013, the rise in tariffs was about 12% above the preceding levels.Many inputs into health service delivery in Ghana are imported. Figure 1 shows the total value of NHIS reimbursements for medicines and services to providers over time. In cedi terms, the amounts have risen steeply, in dollar terms (exchange rates at 4.00 pm UT on 30th June each year) the rise is slower and flattening.

### Other provider payment methods

The health system had other provider payment methods with which the G-DRG for services and itemized fee schedule for medicines came to co-exist. The Government of Ghana line item, global budgets, and salary payments provide supply side subsidies to public providers from consolidated tax funds for service delivery, administration, infrastructure, equipment, tools, and supplies. Some supply side subsides to public sector providers also come from donor Sector Budget Support and program funding. Allocation of Government of Ghana funds to public sector facilities is often based on historical budgets despite the theory that with the Medium Term Expenditure Framework reform these budgets would to be tied to Ministry, Department, and Agency vision, mission, objectives, and plans of action for the year. A major reason for this would appear to be that the national budget is so constrained it makes it difficult to relate allocations to requests. Fund flows also tend to be irregular and unpredictable in amount. CHAG facilities also receive supply side subsidies since a large proportion of staff salaries are paid from Government of Ghana Consolidated Funds. Most CHAG facilities are located in underserved areas, considered as priority for service delivery, and are seen as supporting government to attain its equity and access goals in service delivery.

**Figure 2:**
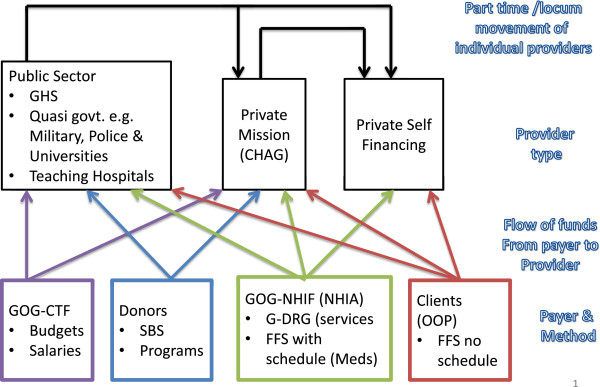
Payment and service provision inter-relationships.

Private self-financing (for-profit) providers receive no government supply side subsidies. They rely for their income on activity-based payments related to services and population, namely out of pocket payments by clients, direct reimbursements by some corporations, and, since 2004, NHIS reimbursements. Sometimes these providers chose not to participate in the NHIS because they consider the tariffs inadequate. Before the introduction of the NHI scheme clients in public and private sectors paid out of pocket fees based on itemized fees with no fee schedule. Non-insured clients continue to pay these fees in both sectors. Some public sector providers earn extra income through part time locums in private facilities. In some instances they may actually own a private practice. Reports of under the table charges by some public sector providers also exist but it is hard to document the extent of the practice.Implementation of a pilot per capita payment method for primary outpatient care has been on-going in the Ashanti region of Ghana, which has 19% of the population, since January 2012. Plans to scale up per capita payment for primary care nationwide have been announced. Figure 2 summarizes the purchasers and providers in the Ghana health system, method(s) used by each purchaser, and fund flow from purchaser to provider.

### Theoretical framework

**Figure 3:**
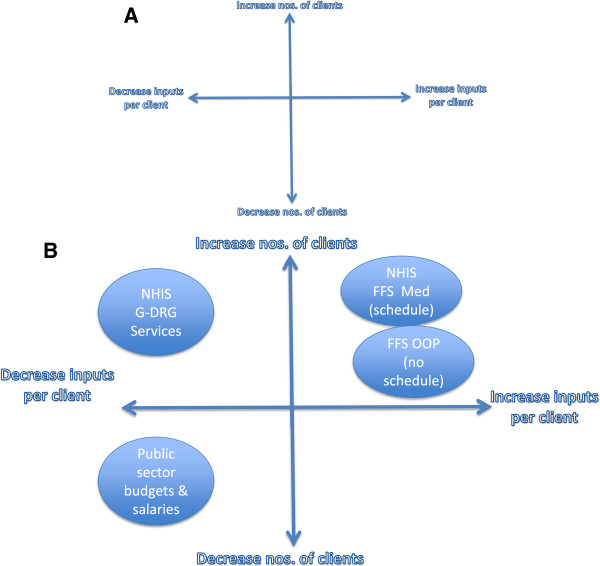
**(A) Dimensions of supply. (B)** Theorized map of provider payment in Ghana onto dimensions of supply incentives.

There are several theories of provider behaviour that predict incentives and supply responses to payment methods. Building on literature reviews [4,5] of provider payment methods and the financial incentives for supply behaviour they potentially generate we theorized that supply can be modified in two broad dimensions. One dimension is related to the numbers of client encounters a provider has in a given time period. This dimension can change by an increase or decrease in the individual numbers of clients making up the provider’s client pool, or in numbers of visits per client for the same pool, or a combination of the two. Question in relation to this dimension would be whether there are incentives for providers to try to increase or reduce numbers of encounters. A variety of means, such as modifying opening and closing hours, referring or not referring clients to other providers, making the service more or less attractive to clients, etc., could be employed by providers to affect this dimension. Incentives to have more client encounters would not be infinite but bounded by the provider infrastructure, equipment, tools, supplies, and human resources, as well as the perceived and actual value of the alternative uses of provider’s time and resources.The second dimension of supply would be related to the inputs into the services provided in each client encounter regardless of the number of encounters. The manifestation of this dimension would be related to incentives to supply more or less medicines, laboratory tests, procedures, etc. Again, the incentive to supply more or less would be bounded rather than infinite. These two dimensions could be simply summarized in the form of a graph as in Figure 3A. The theorized expected incentives in these two dimensions for each of the provider payment methods operational in Ghana, based on the review of the literature, without analysing the effect of context and interactions with other provider payment methods in the system, can be mapped onto this graph as in Figure 3B.

Since our objective was to understand service supply behaviour and incentives related to the NHIS provider payment methods in the context of Ghana and its health system, we went beyond the simple linear theoretical model of Figure 3A,B and drew on realistic evaluation [27] and systems thinking theories including the concept of the health system as a CAS [6] for our analysis. We have already described features of CAS and the relevance to this study in our introduction.Realistic evaluation theory suggests that a particular action(s) leads to outcome(s) or effect(s) by triggering a mechanism or set of mechanisms acting in context. The link between action and outcome is thus complex and non-linear. Thus, the observed effects of provider payment methods in the Ghanaian health system may not manifest as direct linear causative effects between the payment method and the observed service supply behaviour as theorized in Figure 3B. Rather, any given provider payment method will interact with the context and other methods to trigger mechanisms that would lead to observed service supply behaviours and incentives. They could, but would not necessarily be, as predicted from the theoretical linear analysis in Figure 3B.

## Methods

The study was carried out over a 6-month period from March to August 2013 using mixed methods of data collection. Google and PubMed searches for “provider payment methods in Ghana”, “provider payment systems in Ghana”, “Ghana DRG payment system”, and “Ghana National Health Insurance Scheme” were used to identify grey and published literature for review. The Ministry of Health, Ghana Health Service, and NHIA websites were searched for reports with relevant information. Additionally, key informants were asked whether there were any reports, administrative memo, and other material in their records and archives related to provider payment methods that could be made available for review. The search was focused on the period January 2003 to August 2013. Routine management information system data of providers and schemes related to utilization and claims over the same period was obtained for secondary analysis. Some of the national level provider data could not be obtained for the period 2005 to 2007.

Primary data collection at the regional and district level was conducted between April and June 2013. Follow-on interviews and two validation meetings with respondents to discuss our initial analysis and conclusions were conducted in July/August 2013. Table 1 summarizes the geographic location of primary data collection, methods of data collection, and number of respondents for each method employed. The study had several questions beyond the ones presented in this paper. We only describe variables and indicators from which data was drawn to answer the questions of this paper.

**Table 1:** Geographic location of primary data collection (level, facility type, and ownership), method of data collection, and numbers of respondents

**Level (National, Regional, District/Municipal/Sub-metro)**				**Number of respondents**			
	Facility name	Facility type	Ownership	Key informant in-depth interview	Client exit interview	Client waiting time assessment	Client prescription analysis
National	Not applicable	Not applicable	Not applicable	14	0	0	0
Ashiedu Keteke sub-metro	Ussher polyclinic	Polyclinic	Public (GHS)	2	35	35	35
	PML Children’s hospital	Hospital	Public (GHS)	0	33	34	34
	Cathedral clinic	Clinic	Private (PSF)	0	35	25	25
	Ashiedu Ketete district scheme	Purchaser	Public (NHIA)	1	0	0	0
La Dadekotopon sub-metro	La General hospital	Hospital	Public (GHS)	2	35	37	37
Madina La Nkwantang municipality	Pentecost hospital	Hospital	Private (CHAG)	2	35	34	34
	North Legon hospital	Hospital	Private (PSF)	2	31	22	22
	Madina Polyclinic (New Road)	Polyclinic	Public (GHS)	2	35	35	35
	Ga municipal scheme	Purchaser	Public (NHIA)	1	0	0	0
Ada East District	Ada HC	Health centre	Public (GHS)	2	35	35	35
	Pute CHPS	CHPS	Public (GHS)	2	5	4	4
**TOTAL Greater Accra Region**				**30**	**279**	**261**	**261**
Dorma municipality	Amasu HC	Health centre	Public (GHS)	2	10	11	11
	Twumkrom CHPS	CHPS	Public (GHS)	2	0	0	
	Dorma Presby Hospital	Hospital	Private (CHAG)	2	30	23	23
	Saviour clinic	Clinic	Private (PSF)	1	13	13	15
	Dormaa district scheme	Purchaser	Public (NHIA)	1			
Dorma West district	Nkrankwantakrom	Health centre/DH	Public (GHS)	2	29	39	39
	Kojo Kumi Krom	Health centre	Private (CHAG)		29		
	Yaa Krom HC	Health centre	Private (CHAG)	2	16	2	2
	Kwakuanya Ebenezer Methodist	Clinic	Private (CHAG)	1	0	0	
	Kyremesu Presby HC	Health centre	Private (CHAG)	2	3	18	18
	Kwabenadwomo	Health centre	Public (GHS)	2	13	0	
**TOTAL Brong Ahafo Region**				**17**	**143**	**106**	**108**
Sisala East district	Tumu hospital	Hospital	Public (GHS)	2	35	37	37
	Wellembele HC	Health centre	Public (GHS)	2	16	19	19
	Nnamdouonu CHPS	CHPS	Public (GHS)	2	7	8	8
	Mama Mary	Clinic	Private (PSF)	2	18	22	22
	Sissala East district scheme	Purchaser	Public (NHIA)	1	0	0	0
Wa municipality	Kambali HC	Health centre	Public (GHS)	2	30	37	37
	Tampalipani CHPS	CHPS	Public (GHS)	2	4	4	4
	Islamic hospital	Hospital	Private (PSF)	2	35	26	32
	Wa Municipal scheme	Purchaser	Public (NHIA)	1	0	0	0
**TOTAL Upper West Region**				**16**	**145**	**153**	**159**
**GRAND TOTAL**				**63**	**567**	**520**	**528**

GHS, Ghana Health Service; PSF, Private Self-Financing; CHAG, Christian Health Association of Ghana; CHPSC, Community Health Planning and Services Compound; NHIA, National Health Insurance Authority.

The national level key informant interview guide items explored how the G-DRG and itemized fee schedule for medicines payment methods were designed and implemented, and perceptions of service supply incentives and behaviour related to the design and implementation. At the district level, key informant interviews were held with District Insurance Scheme office managers, District Health Directorate staff, and health facility managers. Areas covered in the interviews were observations and perceptions of how the NHIS provider payment system had affected health facility and insurance scheme office decisions related to service supply and the advantages and disadvantages of the methods.

Within health facilities, observations of time spent by clients at different service points and in total, prescription content analysis, and client exit interviews were carried out using observation checklists, interview guides, and semi-structured questionnaires. The client exit interviews had a mix of closed and open ended items to explore client experience in the clinic related to service supply and responsiveness, previous experiences, opinions about the NHIS, and suggestions for making the NHIS more responsive.

### Sampling

Sampling was purposive. Participants for the national level key informant in-depth interviews were selected from the list of designers of the G-DRG payment method [28]. For regional and district primary data collection, we stratified the country into three zones of relatively similar socio-economic characteristics, namely Northern (Upper East, Upper West, and Northern regions); Central (Brong Ahafo and Ashanti regions), and Southern (Volta, Eastern, Greater Accra, Central, and Western regions). Within the Central zone the Brong Ahafo region was purposively selected because the on-going pilot of capitation in the Ashanti region would make it difficult to evaluate incentives inherent in the nationwide payment systems as against capitation pilot effects. Within the Southern ecological zone, the Greater Accra region was purposively selected because of its peculiarity of being 90% urban with a large and active private self-financing provider community and the lowest average poverty levels in the country. Within the three Northern regions, which have the highest percentage of rural populations and poverty levels in the country, the Upper West Region was randomly selected by balloting since there was no clear rationale to justify purposive selection.

Within each of the three regions, a list of the most recent local government demarcation and classification of districts with districts stratified into rural, municipal, and metropolitan was obtained. One district in each category was selected per region by balloting. Greater Accra was the only region with metropolitan districts and a sub-metropolis in Accra was selected by balloting. The NHIA office covering each selected district was included in the sample.

In the selected districts, a list of government, CHAG, and private self-financing facilities was obtained from the Ghana Health Service and one NHIA accredited district hospital, health centre, and CHPS compound in the public sector were selected by balloting. Where the district had CHAG and private self-financing facilities, one CHAG and one private facility were selected by balloting if there was more than one; if there was only one it was selected. During data collection, some selected facilities had to be substituted with the nearest facility of the same category because the information in the national level facility listings did not always reflect what was happening at the frontline and the selected facility was no longer functional.

Data collectors visited each clinic starting at the morning shift hours of 8.00 am. All clients entering the clinic – regardless of insurance status – were tracked for time spent at the different service points until a maximum of 35 clients was reached. Some of the smaller clinics had low client loads and it was not possible to get 35 clients in one day but the time frame and budget of the study did not allow repeat visits. Prescriptions issued to these clients were copied for analysis, and an exit interview administered. Ethical clearance was obtained from the Ghana Health Service Research and Development division; all tracking and interviewing was done with informed consent.

### Data analysis

The study was carried out in response to a request by the Ghana NHIA for an evaluation of its DRG payment methods. The constraints of the time frame of the request meant that the data collection and analysis was done using overlapping processes. The team had mixed qualitative and quantitative data collection and analysis skills among the members. The same two members of the research team did all the national level in-depth interviews and, together with a third team member, they performed the qualitative analysis. For the district level primary data collection, the research team split into three groups to collect data with support from research assistants.

Apart from notes during the interview, in-depth interviews were recorded and transcribed. Analysis was manual to identify themes, commonalities, and contrasts. The open-ended questions in the exit interviews were typed into Excel, coded, and classified by themes and sorted for analysis. Primary quantitative data analysis was done in Excel and Stata. Routine health management information system data was analysed in Excel. We used frequencies, cross tables, and trend lines for the quantitative data analysis. Team members were assigned responsibility for analysing particular data sets depending on their expertise.

To help generate a more holistic theory as to the relationships between provider payment methods, service supply incentives and behaviour, and what mechanisms explain these observed effects, we used causal loop and causes tree diagrams [29] – both systems thinking tools.

### Validity, quality assurance, and limitations

Several methods were used to ensure validity. Firstly, we have presented our methods in detail to enable readers to judge the quality of the data. Secondly, the whole team discussed analysis and conclusions from each data set, and findings from different data sets related to the same question were compared as part of triangulation and minimizing individual team member biases. This also allowed a more reflexive approach to data analysis. Thirdly, we paid attention to extreme as well as middle cases in our analysis and did not focus on frequently repeated responses only. Fourthly, before finalizing our report, we organized two different half-day validation meetings with representatives of our respondents to present our initial analysis and conclusions and obtain their feedback. This was part of the iterative process of data collection and data analysis. We also made our draft report available to respondents who were willing to read it, to check if it was valid from the perspective of their experiences that we were trying to describe and analyse.

### Findings and discussion

**Figure 4:**
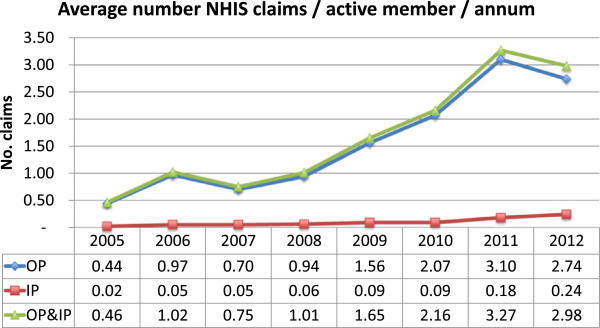
Outpatient (OP) and inpatient (IP) claims per active member per annum (NHIA routine management information system data).

**Figure 5:**
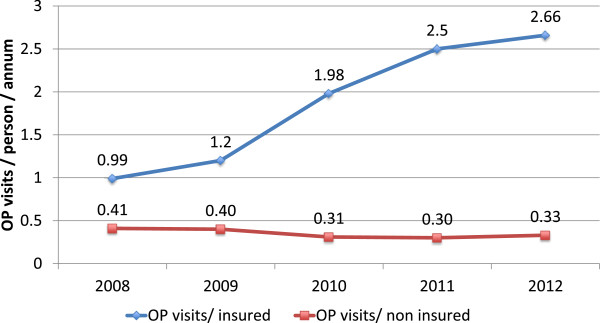
Provider routine management information system data trends in outpatient (OP) visits for insured and uninsured.

**Figure 6:**
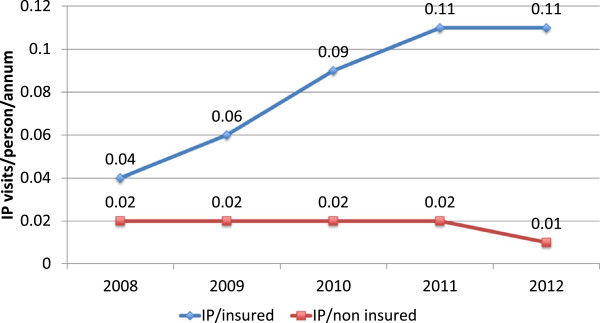
Provider routine management information system data trends in inpatient (IP) visits insured and uninsured.

We used trends in utilization for insured and uninsured to assess changes in supply related to the number of clients seen by providers. The data is summarized graphically in Figures 4, 5, and 6. Both the provider and purchaser data sets tell the same story of increased numbers of visits per active insured member for inpatient and outpatient services. There does not appear to have been a similar change over time in the number of visits to formal providers for the uninsured. Data was not available to enable an assessment and comparison of trends in visits to non-formal providers.

The insurance status of the clients participating in our exit interviews showed the same pattern of a dominance of utilization of formal services by the insured. Of the total of 567 clients in the exit interviews, 432 (76%) had a valid insurance ID card.

It is, however, difficult to come to any firm conclusion from the data available that this utilization reflects incentives for providers to preferentially see insured clients or is due to service provider (supplier)-induced demand. There are several possible reasons related to demand as well as supply that could explain the data. These include the increases in utilization among the insured reflecting increased client demand induced by the removal of the financial barrier and frivolous use by insured clients also related to the removal of the financial barrier. It could, however, also reflect some supplier-induced demand. Several respondents in our national level qualitative interviews mentioned that, for any given illness episode, the G-DRG design allows a provider to bill for three visits for outpatient care – the initial visit and two follow-up visits. It could be to the financial advantage of the provider to bill routinely for all three visits regardless of whether the client needed or even made them.

Making it even more difficult to conclude on supplier-induced demand as a major reason for the increased numbers of encounters per insured client is that our key informant interviews with frontline providers suggested that the G-DRG is leading to some shifting of cases in the form of referral. The quote below illustrates this as well as the impression we got that there was a disincentive to see certain kinds of insured clients because providers felt the reimbursements were inadequate for the inputs needed to manage the case.

*“…you can imagine somebody bringing an ulcer… you know that (dressing) a big sore daily… the cost will go up so you will lose… so we were losing, so that was why most of us were not dressing this thing, we refer them to the hospital… yes, even the suturing too was a problem; the money was just a token.”* Rural Health Centre nurse

The actual as well as perceived inadequacy of the reimbursement rates were compounded by the delays in reimbursements. To illustrate with the words of a hospital medical superintendent:

“…The payment system has really broken down to a certain extent. They are not consistent with the payments and it is disturbing our work. It makes us financially not sound… Promptness is the bigger problem rather than the rate… If they would pay us promptly I would be so happy.”

These observations lead to other findings on the dimension of supply related to input use per client. The indicators explored to help understand this dimension of provider supply decisions were volume of tests and procedures, medicines prescribing, and client time spent in facilities.

### Volume of diagnostic tests and procedures

Some of the responses obtained from providers and clients suggest that the bundled payments of the G-DRG for services were a disincentive to carry out extensive diagnostic investigations whether they were needed or not, for example:

*“…the grouped billing… is a disincentive to carry extensive investigations”* Pharmacist, Urban Polyclinic

An NHIS subscriber described how he presented for services without showing his insurance card. After his history and examination, he was asked to do several laboratory tests to help confirm a diagnosis. At this point, he mentioned he had an insurance card and asked if that could cover his treatment including the laboratory tests. There was a subtle change in the facial expression of the staff and he was asked why he had not provided this information earlier. He was then asked to please give his folder back for review and wait. After a while, the folder was brought back to him with the laboratory tests cancelled, and the information that they were not needed. He could just go and collect his medicines.

### Medicine prescribing

**Figure 7:**
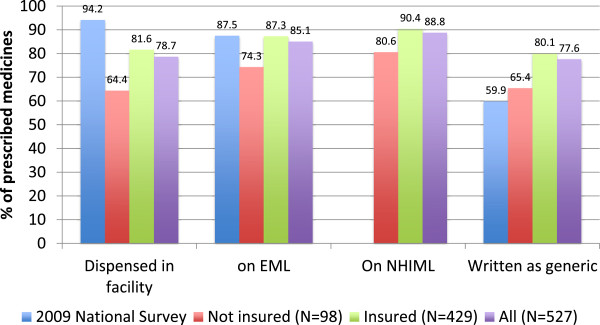
Provider medicine supply behaviour (prescribing and dispensing).

The literature review of the incentives associated with different payment methods suggests that over or at least adequate provision would be an incentive for the supply of medicines under the NHIS, given that an itemized fee for medicine schedule is the payment method. Our data sometimes suggested, but was not always convincingly in support of, a situation of adequate or over rather than under provision of medicines. The average number of medicines per outpatient prescription in our sample was four for all prescriptions (n = 527)^a^, three for the non-insured (n = 98), and four for the insured (n = 429). The most recent national data on prescribing indicators available for comparison was the pharmaceutical situation assessment carried out in May/June 2008 [30]. It unfortunately did not compare data between insured and uninsured. The average number of medicines per prescription in this survey was four.Other prescribing indicators from our survey are summarized in Figure 7 and compared with data from the 2008 national survey. There was no data on whether medicines were on the NHIML in the 2008 survey. It would be expected that facilities would supply nearly 100% of the medicines prescribed from their dispensaries since, in theory, the more medicines supplied, the more income the provider earns. In our survey, however, only 78.7% of medicines prescribed were dispensed in the facility. A higher percentage was dispensed in the facility to the insured (81.6%) as compared to the uninsured (64.4%). However, as Figure 7 shows, compared to the 2008 national survey data where 94.2% of all medicines prescribed were dispensed in the facility, the percentage of medicines prescribed that were dispensed in the facility, whether to insured or uninsured, is low. It raises questions as to whether something other than insurance status and provider payment method is modifying prescribing and dispensing behaviour.The NHIS policy requires that medicines are prescribed by generic name and are on the NHIML, otherwise they do not qualify to be supplied “free” under the NHIS. As Figure 7 shows, there was higher prescribing of medicines by generic name and from the NHIML for insured as compared to uninsured clients. It is reasonable to suspect that this is an influence of the provider payment method and its associated rules. However, in spite of these rules, not all medicines for insured clients were prescribed by generic name or listed on the NHIML. It would appear that something else is also influencing behaviour. The influence is probably bigger for the uninsured who do not have the effect produced by the rules of the NHI provider payment method. Our data does not allow a complete explanation of what is causing these prescribing and dispensing behaviour gaps, which are wider for the uninsured than the insured. We can only make some guesses based on our observations. One of them is that providers repeatedly complained that the reimbursement rates for some medicines on the NHIML are too low. Perhaps, the actuality as well as the perception of too low tariffs could negate, in part, any incentives to prescribe and dispense such medicines. Secondly, weaknesses in the implementation of the rational use of medicines policy in Ghana could also explain the uninsured data. The rational use of medicines policy requires that all medicines are prescribed by generic name and are listed on the Essential Medicines List. The Essential Medicines List and the NHIML overlap, but not completely. Finally, the condition of some patients may just have required medicines to be prescribed outside the NHIML and the Essential Medicines List, and which were not available as generic. It is doubtful, however, if such cases at the outpatient primary care level should account for as many as 10 to 25% of prescriptions.

The exit interview data confirmed the failure to supply all medicines prescribed in the facility dispensary and also showed insured client discontent with this failure. Of the 41 clients in the exit interviews (7% of the total sample) who felt the NHIS was bad, a little over half (22) gave a reason related to the failure to supply all medicines prescribed free as part of their NHIS benefits. Examples typical of these responses include:

“The aspect where the scheme does not cover all the drugs is worrying to us especially we the poor…”

“They do not give all the drugs”

“Buying drugs outside the hospital while you still have a valid insurance”

“…dislike the NHIS because initially it was supposed to be free but now I’m made to buy drugs anytime”

Some out of pocket payments by insured clients is not a new finding; as many as 94% of respondents in the Ghana Demographic and Health Survey [13] reported sometimes making out of pocket payments for medicines, services, or both. The SHINE project also documented insured client out of pocket payments. They were, however, significantly lower than uninsured client payments [31,32]. Some of these out of pocket payments are for services and medicines not covered by the NHIS. Others are related to managerial inefficiencies, e.g., stock outs, and others to reluctance to stock and supply NHI clients with items that are seen as potentially causing financial loss to the provider because of unattractive NHI tariffs.

### Time spent with patients

**Figure 8:**
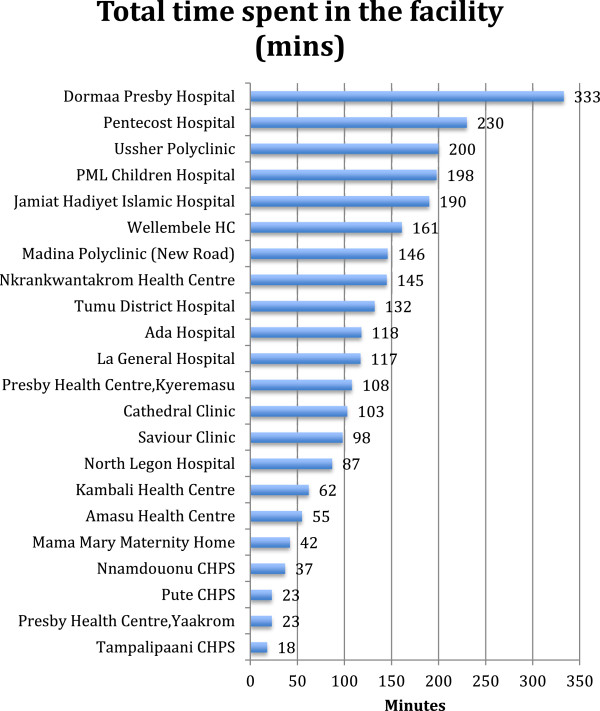
Total time spent by facility.

It was not clear that time spent in the clinics by clients was related to provider payment incentives. Client loads and staffing constraints rather appeared to be the influences over it. Figure 8 summarizes total time spent by facility. The longest waiting times were in the crowded mission (CHAG) and public (Ghana Health Service) hospitals, polyclinics and health centres. The private hospitals and clinics and the CHPS compounds were we recorded the lowest times spent by patients in getting care were also the facilities in which we observed lower client numbers.

### Pulling it all together

**Figure 9:**
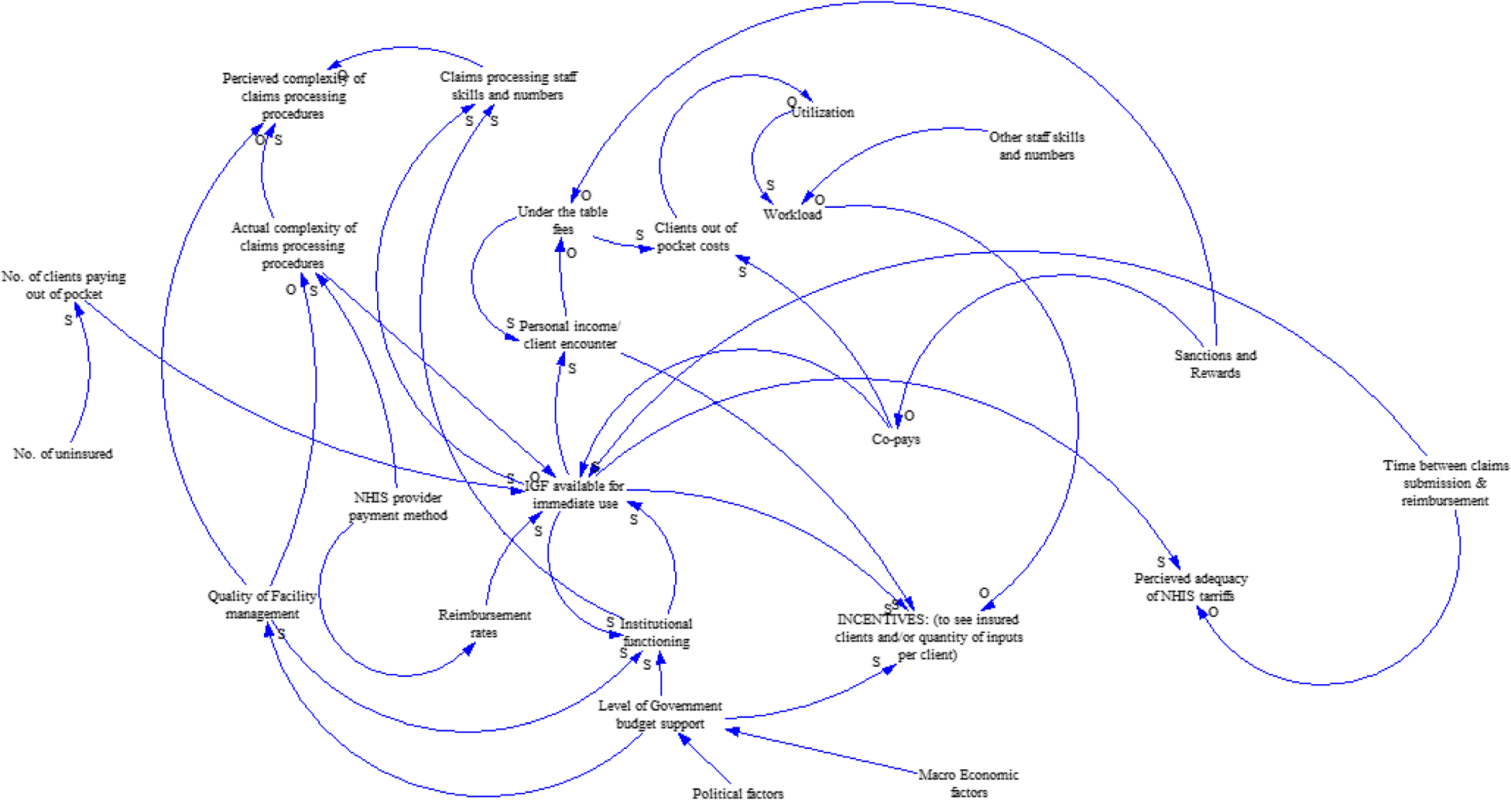
Causal loop diagram.

We have qualitatively explored some answers to questions related to the “what” and the “why” of service supply behaviour and incentives related to provider payment methods of the Ghana NHIS. To answer the “what” question, we have conceptualized and described service supply behaviour in two dimensions of numbers of insured clients and inputs into management of individual clients seen. To answer the “why” question, we have drawn upon systems thinking and realist evaluation theories and examined the wider national context, the health system, and their influence. We now pull these pieces together to generate potential explanatory theory employing the systems thinking tool of causal loop and causes tree diagrams qualitatively. Figure 9, a causal loop diagram, is our concluding theoretical model. Since the diagram is qualitatively constructed, it provides no indication of magnitude of effect.

As for any causal loop diagram the theorized direction of influence of one factor on the other is show by the direction of the arrow. The labels at the tips of the arrows indicate whether the influence is to cause a decrease or increase in the level of the influenced factor. An “S” sign against the arrow head means that, as the causal variable increases or decreases, the influenced variable changes in the same direction. An “O” sign means the change is in the opposite direction. Where there is neither an “O” or an “S” sign, the relationship is something other than a straight forward increase or decrease.

**Figure 10:**
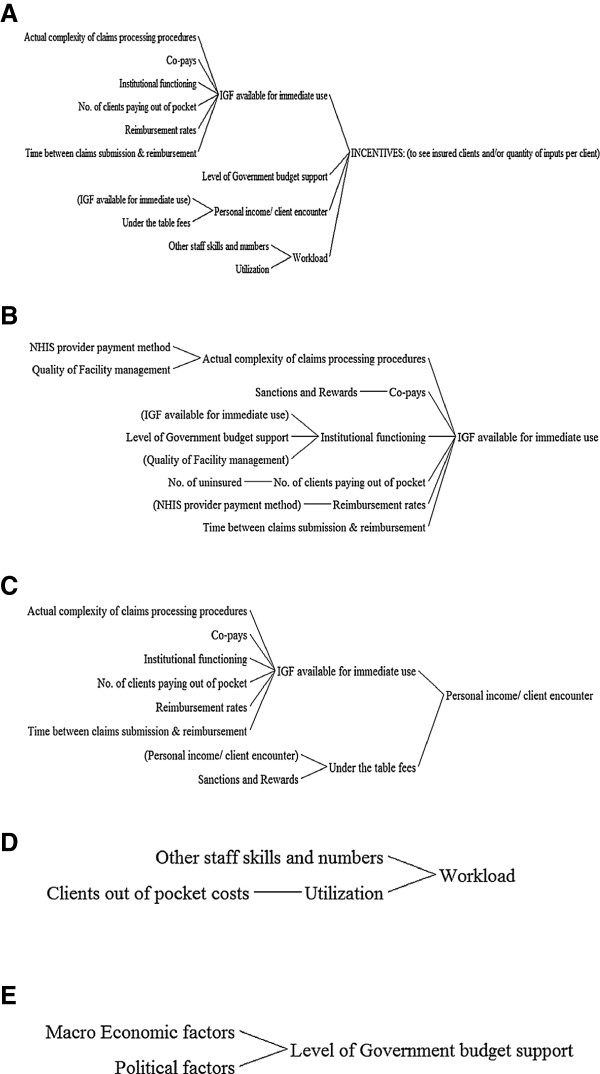
**(A) Factors influencing service supply incentives. (B)** Factors influencing IGF available for immediate use. **(C)** Factors influencing personal income per client encounter. **(D)** Factors influencing workload. **(E)** Factors influencing level of direct government budget support to providers.

Thus, for example in Figure 9, we theorize that the amount of internally generated funds (IGF) available for immediate use through NHI payments influences incentives to supply service to insured clients. IGF is a term used in Ghana to describe funds that are generated and retained for use at the facility level as compared to ‘external’ funds such as Central Government allocations. IGF comes from NHI payments, out of pocket fees, and, in a few instances, reimbursements from corporations and agencies on behalf of their workers. For private sector facilities it is their entire source of income. For public sector facilities it forms 80% or more of their income for recurrent expenditure [33]. The more IGF is, the more incentivized providers are to supply services to insured clients in both dimensions. Out of pocket payments have an immediate effect on IGF availability unlike NHIS payments whose effects are modified by the time lag between claims submission and claims payment. Additionally, IGF availability from insured clients is affected by the perceived and actual complexity of claims processing procedures of provider and purchaser. Complex procedures tend to take more time to fulfil and can increase the time lag to final payment. They also require more skilled staff numbers and time and may be more likely to lead to mistakes in claims submission by providers as well as auditing by the purchaser that lead to denial of claims.Causal loop diagrams are difficult to follow for those who have not been involved in the details of constructing them. To make the causal loop diagram easier to follow, we have unpacked its core into a series of cause tree diagrams in Figures 10A–E. Figure 10A shows factors that we theorize from our observations to have a direct influence on supply incentives. These are IGF available for immediate use, level of general budget support provided by government for service delivery, personal provider income per client encounter, and workload. Factors that directly influence each of these factors are unpacked in cause tree diagrams 10B–E.

There does not appear to be a linear relationship between provider payment method and incentives to see more active NHIS card holders or provide more inputs per client. The relationships are indirect and modified by contextual factors. Some of the different factors are controlled by different agents or actors, with some controlled by multiple agents. A reinforcing loop in the causal loop diagram is the link between institutional functioning and IGF available for immediate use. We theorized that better-managed facilities might be better able to find ways of coping with the resource constraints of the health system. Improved resource availability was likely to reinforce better management.

The observed status of many of the variables would produce a tendency to prefer out of pocket paying clients to clients who are paid for by insurance and to contract some but not all service inputs to insured clients. Our data does not allow us to answer the question of whether the current levels of inputs per client are adequate. More services do not necessarily translate into high quality and responsiveness. However, it is doubtful if a tendency to incentivise contraction of service inputs in a system of resource scarcity will ensure high quality and responsiveness.

## Conclusions

In our study setting, service supply behaviour and the incentives driving it cannot necessarily be predicted in abstract from the theory about the anticipated response to a given payment method. The wider national context as well as characteristics of the health system into which the method is introduced shape and modify supply behaviour and incentives. This is not surprising given that the payment method reforms have been introduced into a complex adaptive system. The individual agents (whether institutions, persons or groups) in such systems are interconnected and have the freedom to act in ways that are not always predictable. Whether ignored or acknowledged, complexity remains and affects outcomes in such systems. To be able to cope, it is better to recognize, understand and try to work with complexity rather than engage in the futile effort of trying to “reduce” it with linear approaches [34,35].

Provider payment reform in low- and middle-income countries should pay at least as much, if not a little more, attention to context of the reform and the potential interactions between the reform and context and the resulting intended and unintended effects as to the method itself in the design and implementation of reform.

Finally, Gauri [36] has observed that “*data limitations, selection effects and numerous confounding variables*” make study difficult and limits the availability of empirical research on provider payment mechanism effects on providers in low- and middle-income countries. We agree from our experience and make a plea for continued work on methodological approaches in such settings.

### Endnote

^a^Prescriptions transcribed with missing data dropped from the analysis accounts for the difference between the total number of prescriptions in the sample (528) and the number of prescriptions from which analysis is presented (527).

## Abbreviations

CAS: Complex Adaptive Systems; CHAG: Christian Health Association of Ghana; CHPS: Community Health Planning and Services; G-DRG: Ghana Diagnostic Related Groupings; IGF: Internally Generated Funds; NHI: National Health Insurance; NHIA: National Health Insurance Authority; NHIML: National Health Insurance Medicines List; NHIS: National Health Insurance; SSNIT: Social Security and National Insurance Trust; UHC: Universal Health Coverage.

## Competing interests

The authors declare that they have no competing interests.

## Authors’ contribution

All authors contributed to study design, data collection, and analysis. IAA conceptualized and wrote the paper. All authors read and commented on the draft. All authors read and approved the final manuscript.
